# Identification and Validation of a QTL for Bacterial Leaf Streak Resistance in Rice (*Oryza sativa* L.) against Thai *Xoc* Strains

**DOI:** 10.3390/genes12101587

**Published:** 2021-10-09

**Authors:** Tripop Thianthavon, Wanchana Aesomnuk, Mutiara K. Pitaloka, Wannapa Sattayachiti, Yupin Sonsom, Phakchana Nubankoh, Srihunsa Malichan, Kanamon Riangwong, Vinitchan Ruanjaichon, Theerayut Toojinda, Samart Wanchana, Siwaret Arikit

**Affiliations:** 1 Plant Breeding Program, Faculty of Agriculture at Kamphaeng Saen, Kesetsart University, Nakhon Pathom 73140, Thailand; tripop.t@ku.th; 2 National Center for Genetic Engineering and Biotechnology (BIOTEC), National Science and Technology Development Agency (NSTDA), Khlong Luang, Pathum Thani 12120, Thailand; wanchana250736@gmail.com (W.A.); sattayachiti65@gmail.com (W.S.); yupinmomay@gmail.com (Y.S.); p.nubankoh@gmail.com (P.N.); vinitchan.rua@biotec.or.th (V.R.); theerayut@biotec.or.th (T.T.); 3 Rice Science Center, Kasetsart University, Kamphaeng Saen Campus, Nakhon Pathom 73140, Thailand; mutiarakp@gmail.com; 4 Department of Plant Pathology, Faculty of Agriculture, Kasetsart University, Bangkok 10900, Thailand; srihunsa.m@ku.th; 5 Department of Biotechnology, Faculty of Engineering and Industrial Technology, Silpakorn University, Sanamchandra Palace Campus, Nakhon Pathom 73000, Thailand; kanamonnueng@gmail.com; 6 Department of Agronomy, Faculty of Agriculture at Kamphaeng Saen, Kasetsart University, Kamphaeng Saen Campus, Nakhon Pathom 73140, Thailand

**Keywords:** QTL-seq, *Xanthomonas oryzae* pv.*oryzicola*, *Xoc*, bacterial leaf streak (BLS), resistant gene, *xa5*, rice, MAS, molecular breeding

## Abstract

Rice is one of the most important food crops in the world and is of vital importance to many countries. Various diseases caused by fungi, bacteria and viruses constantly threaten rice plants and cause yield losses. Bacterial leaf streak disease (BLS) caused by *Xanthomonas oryzae* pv. *oryzicola* (*Xoc*) is one of the most devastating rice diseases. However, most modern rice varieties are susceptible to BLS. In this study, we applied the QTL-seq approach using an F_2_ population derived from the cross between IR62266 and Homcholasit (HSC) to rapidly identify the quantitative trait loci (QTL) that confers resistance to BLS caused by a Thai *Xoc* isolate, SP7-5. The results showed that a single genomic region at the beginning of chromosome 5 was highly associated with resistance to BLS. The gene *xa5* was considered a potential candidate gene in this region since most associated single nucleotide polymorphisms (SNPs) were within this gene. A Kompetitive Allele-Specific PCR (KASP) marker was developed based on two consecutive functional SNPs in *xa5* and validated in six F_2_ populations inoculated with another Thai *Xoc* isolate, 2NY2-2. The phenotypic variance explained by this marker (PVE) ranged from 59.04% to 70.84% in the six populations. These findings indicate that *xa5* is a viable candidate gene for BLS resistance and may help in breeding programs for BLS resistance.

## 1. Introduction

Rice plays an important role in global food security, being a staple food for more than half the world’s population. Rice is susceptible to several diseases caused by bacteria, viruses and fungi [1]. Farmers lose an average of 37% of their rice yields to pests and diseases, with losses ranging from 24% to 41% depending on the production circumstances [2]. Two important rice diseases caused by bacteria, bacterial leaf blight (BLB) and bacterial leaf streak (BLS), are common in tropical and temperate regions [3]. BLB is one of the most devastating rice diseases, and BLS is growing more prevalent. Both BLB and BLS are caused by the Gram-negative bacterial pathogen *Xanthomonas oryzae*, but with different pathovars, as BLB is caused by *X. oryzae* pv.*oryzae* (*Xoo*) and BLS is caused by *X. oryzae* pv.*oryzicola* (*Xoc*). Although closely related, *Xoo* and *Xoc* infect rice in different ways: *Xoo* invades the xylem, while *Xoc* colonizes the leaf mesophyll [4]. Compared to BLB, BLS is less destructive and less widespread, and the yield losses caused by BLS are lower. However, in favorable conditions, BLS can spread swiftly and do enormous damage [4]. There are not many reports that have clearly stated the exact damage that can be caused by the disease. However, the approximate yield loss caused by BLS is in the range of 8–32% [5].

The occurrence of the disease is largely confined to tropical and subtropical Asia, including Thailand, Indonesia, Bangladesh, Malaysia, Vietnam and India, in addition to southern China and the Philippines [6]. BLS also occurs in regions of northern Australia [4] and has become a significant problem in parts of Africa [7]. Recently, BLS has become a major problem, especially in Asia and Africa, where cases have been reported quite frequently [4]. In China, where hybrid rice varieties are susceptible to the pathogen, the incidence of BLS is increasing [6]. Despite the seriousness of disease, a coherent system for combating the pathogen has not proven effective. The most effective strategy to control BLS is to grow resistant varieties. However, most rice germplasm, especially Asian rice, is susceptible to BLS [8]. Therefore, breeding broad-spectrum and durable disease-resistant varieties is necessary. Rice resistance to BLS is a quantitatively inherited trait with an unknown molecular mechanism [9]. To date, more than 20 quantitative trait loci (QTLs) have been identified to confer BLS resistance in rice [9,10,11]. Most QTLs and genes identified in association with *Xoc* have been studied in Asian *Xoc* strains, particularly from the Philippines and China, and alongside some African strains [9,10,11,12]. 

Plant genomics has benefited significantly from next-generation sequencing [13], which has enabled the construction of reference genomes for a variety of model and crop species. Combined with high-quality re-sequencing, these data provide a great opportunity to expand the availability of single-nucleotide polymorphism (SNP) data for genomics-based studies, such as genome-wide association studies (GWAS) and QTL-seq, to identify quantitative trait loci (QTLs) or genes associated with multiple agronomic traits [13]. QTL-seq combines the potential of bulk-segregant analysis (BSA) with the power of high-throughput whole-genome sequencing to identify genomic regions with contrasting patterns of a single-nucleotide polymorphism (SNP) index in two bulk populations, each of which containing 20-50 individual plants with extreme phenotypes [14]. Numerous QTLs and genes associated with various phenotypes have been successfully identified using the QTL-seq approach in a variety of crops, including rice [14,15,16,17,18,19,20,21], soybean [22,23], chickpea [24,25], tomato [26] and groundnut [27,28,29].

Although BLS has been confirmed as an important disease in Asia, especially in the tropical and subtropical regions, not many studies on BLS resistance gene/QTL identification have been reported from Thailand. In a recent study, 12 QTLs associated with Thai *Xoc* strains were identified using the GWAS approach [30]. Two QTLs on chromosomes 2 and 5 have been proposed as promising QTLs for broad-spectrum resistance to BLS cuased by Thai *Xoc* strains. However, these identifed QTLs have not yet been validated in a biparental population for efficacy when used in a breeding program. In this study, we applied the QTL-seq method to rapidly identify a QTL conferring resistance to BLS caused by a Thai *Xoc* strain using an F_2_ population segregating for BLS resistance, and developed a molecular marker specific for the candidate gene identified within the QTL that can be used for molecular marker-assisted selection (MAS). The most associated region was located on chromosome 5 toward the beginning of the chromosome, suggesting that *xa5* located in this region is a potential candidate gene for BLS resistance to Thai *Xoc* strains. Based on the detected region, a Kompetitive Allele-Specific PCR (KASP) marker for BLS resistance was also developed based on *xa5*, and was validated in six different F_2_ populations inoculated with another Thai *Xoc* isolate. Because the phenotypic variance explained by this marker (PVE) is high, it could be useful for the marker-assisted selection of BLS resistance in rice breeding programs.

## 2. Materials and Methods

### 2.1. Plant Materials and Growing Condition

For QTL-seq analysis, 433 F_2_ lines from the cross between IR62266, a bacterial leaf streak (BLS) resistant cultivar, and Homcholasit (HCS), a BLS-susceptible cultivar, were used. For marker validation, 450, 461, 441, 453, 358 and 441 F_2_ individuals from the crosses (IR62266 × HCS), (MNTK75 × HCS), (DV85 × HCS), (IR62266 × RD47), (MNTK75 × RD47) and (DV85 × RD47) were used, respectively. To evaluate the resistance of BLS in each population, seeds were grown in a seedling tray in a growth block filled with water to a depth of 5 cm under greenhouse conditions at the Rice Science Center, Kasetsart University, Kamphang Saen Campus, Thailand, at an average temperature of 37 °C for 21 days.

### 2.2. Inoculum Preparation and Phenotyping

For QTL-seq analysis, *Xanthomanas oryzae* pv.*oryzicola* (*Xoc*) isolate SP7-5, one of the representatives of Thai *Xoc* isolates [30], was used for the inoculation of the F_2_ population (IR62266 × HCS). For marker validation, another *Xoc* isolate, 2NY2-2, was used for the inoculation of the six F_2_ populations. The *Xoc* isolates used for inoculation were kindly provided by Assistant Professor Sujin Patarapuwadol of the Department of Plant Pathology, Faculty of Agriculture at Kamphaeng Saen, Kasetsart University. To prepare the inoculum, the bacteria were grown on peptone sucrose agar (PSA) media at 30 °C for 3 days or until complete colonization of the plate by the bacteria. Bacterial suspension was prepared by mixing the bacteria in sterile water to achieve a concentration of 10^8^ cfu/mL (OD_600_ = 0.25). For bacterial inoculation, 200 mL of the bacterial suspension was sprayed with a hand sprayer onto the leaves of 21-day-old plants grown in plastic trays. After inoculation, each tray was placed in a plastic box with a covered lid and incubated overnight. The tray was then removed from the box and kept in the greenhouse with a misting system to control the humidity (75% relative humidity). Fourteen days after inoculation, BLS disease assessment was performed using the International Rice Research Institution (IRRI) Standard Evaluation System (SES) as previously described [30], with the following scale: 0, no lesions observed; 1, small brown specks of pin-point size or larger brown specks with no sporulation center; 3, lesion type the same as in scale 2 but with a significant number of lesions on the upper leaves; 5, typical lesions infecting 4–10% of the leaf area; 7, typical lesions infecting 26–50% of the leaf area; and 9, more than 75% of the leaf area is affected (Figure S1).

### 2.3. Heritability of the Trait

The heritability of BLS resistance in the population (HCS × IR62266) was calculated using the formula previously described [31]. The equation is as follows.

*H_b_*^2^ = [V_F2_ ‒ 1/3 (V_P1_ + V_P2_ + V_F1_)]/V_F2_

where *H_b_*^2^ = broad-sense heritability, V = variance of each generation.

### 2.4. Sample Bulking, DNA Isolation and Whole-Genome Resequencing 

Samples from two extreme phenotype groups were selected for sample bulking. From the 433 samples of the F_2_ population (IR62266 × HCS), the 50 individuals that exhibited the most resistant phenotype and the other 50 individuals that exhibited the most susceptible phenotype were used to generate the resistant bulk (R-bulk) and susceptible bulk (S-bulk), respectively. High-quality genomic DNA was isolated from the leaves of each individual in the resistant and susceptible groups and from the parent cultivars IR62266 and HCS using a DNeasy Plant Mini Kit (Qiagen, Hilden, Germany). The R-bulk and S-bulk were then prepared by mixing an equal amount of DNA from each individual in each group. The DNA samples were sent to Novogene Co., Ltd. (Hong Kong) for DNA-seq library construction and whole-genome sequencing. The DNA-seq library was sequenced using an Illumina HiSeq 2500 platform (Illumina, San Diego, CA, USA) to generate the 2 × 150 bp paired-end read data with a sequencing depth of approximately 20× the rice genome (~373 MB) for each sample.

### 2.5. Data Analysis Via the QTL-Seq Pipeline and Candidate Gene Annotation

QTL-seq analysis was performed using a QTL-seq pipeline as described previously [14,32]. A reference genome of the parent, IR62266, was created using the pipeline and was used as the reference for read mapping of the two bulk samples. The reads of IR62266 were first aligned on the public reference genome (Nipponbare: IRGSP1.0) using the Burrows–Wheeler Aligner (BWA) [33]. Then, the variants representing the IR62266 parent substituted the bases in the Nipponbare reference genome. The clean reads from each bulk were then mapped onto this IR62266 reference genome. SNP calling and SNP index calculations were performed using the QTL-seq pipeline as previously described [14,32]. “SNP-index = 1” indicates that reads are derived only from the HCS genome, whereas “SNP index = 0” indicates that the reads are derived only from IR62266 genome. An SNP index of 0.5 indicates an equal genome contribution from both parents. A sliding window analysis was performed by averaging the Δ(SNP index), and the plots of the distribution of average SNP index and Δ(SNP index) compared between the two bulks were generated. The candidate genes within the detected regions were obtained based on the Rice Annotation Project database (RAP-db: https://rapdb.dna.affrc.go.jp, accessed on 7 September 2021).

### 2.6. Development of a KASP Marker and Validation in Populations with Different Genetic Backgrounds

A Kompetitive Allele-Specific PCR (KASP) marker for *xa5* was developed based on the two consecutive SNP variants at the positions 437,499 and 437,500. The KASP assays were performed based on the manual of LGC Genomics (http://www.lgcgenomics.com, accessed on 7 September 2021). The KASP reaction was performed in a 96-well format and set up as 5 µL reactions with 2 µL of DNA template, 0.075 µL of assay mix and 2.5 µL of master mix. Amplification was started at 94 °C for 5 min, followed by 10 cycles at 94 °C for 20 s and at 61 °C for 60 s (touchdown to 61 °C, decrease of 0.6 °C per cycle), followed by 27 cycles at 94 °C for 20 s, 55 °C for 30 s and a rest step at 37 °C for 1 min. After amplification, the fluorescence signals from the end PCR products were read with the QuantStudio 6 Flex Real-Time PCR System (Thermo Fisher Scientific, Watham, MA USA). For genotyping, the KASP marker was used to genotype in six different F_2_ populations. A single-marker analysis was performed by the lm() function in R (http://www.r-project.org, accessed on 7 September 2021) using the genotype data from the KASP marker and the phenotypic data of 96 lines in each F_2_ population.

## 3. Results

### 3.1. Phenotype of F_2_ Population and the Construction of Resistant and Susceptible Bulks

**Figure 1:**
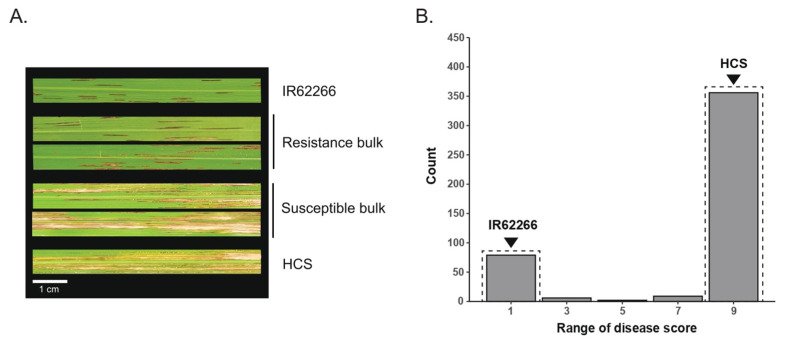
Bacterial leaf streak (BLS) phenotype in parental lines and F_2_ plants. (**A**) The BLS symptom in the resistant parent (IR62266) and susceptible parent (HCS), and in the representative F_2_ lines in the resistant bulk and susceptible bulk. (**B**) Distribution of BLS scores in 433 F_2_ lines. The scores of the resistant parent and susceptible parent are indicated by a triangle. The dashed rectangle indicates the plants that were selected to produce the resistant bulk and susceptible bulk.

Bacterial leaf streak (BLS) resistance screening was performed on 433 individuals of the segregating F_2_ population from a cross between IR62266 and Homcholasit (HCS) against the SP7-5 isolate of Thai *Xanthomonas oryzae* pv.*oryzicola* (*Xoc*). Homcholasit is a submergence-tolerant indica rice variety developed by Rice Gene Discovery, BIOTEC, Thailand, with good cooking quality but high susceptibility to BLS. IR62266 is an indica rice variety developed by the International Rice Research Institute (IRRI) and used as a source of BLB resistance [34,35]. This rice variety also has high resistance to several Thai *Xoc* isolates and has been recommended as a potential source of BLS resistance [30]. The BLS disease was evaluated 14 days after inoculation using the leaf lesion scoring index (with scores from 1 to 9 indicating highly resistant and highly susceptible, respectively). The results clearly show a different phenotype between the resistant parent, IR62266, and the susceptible parent, Homcholasit (HCS), as the BLS symptom was less pronounced in IR62266 (a value of 1) than in HSC (a value of 9) (Figure 1A). The BLS disease scores among the 433 F_2_ plants showed a bimodal distribution, demonstrating that the majority of F_2_ plants (339 lines; 78.29%) were susceptible to BLS, with a severity score of 9. In addition, a smaller group of 77 F_2_ plants (17.78%) was resistant to BLS, with a severity score of 1 (Figure 1B; Table S1). There were also F_2_ plants with an intermediate score, including six, two and nine plants with scores of 3, 5 and 7, respectively. The broad sense heritability of the BLS resistance scores in the greenhouse condition, estimated by the variance in the different generations, was high (*H_b_*^2^ = 0.98). This indicates that resistance to the *Xoc* isolate SP7-5 in this rice cross was strongly inherited and possibly controlled by recessive inheritance. Based on the BLS resistance scores, we selected 50 F_2_ individuals with high resistance to BLS (score of 1) and another 50 individuals with high susceptibility to BLS (score of 9) to form the resistant bulk (R-bulk) and susceptible bulk (S-bulk), respectively (Figure 1; Table S1).

### 3.2. Whole-Genome Re-Sequencing of the Two Bulks and Parents

Whole-genome re-sequencing data were generated from the resistant bulk (R-bulk), susceptible bulk (S-bulk), and parental strains HCS and IR62266 using the Ilumina Hiseq 2500 platform. The total number of short reads (150 bp long) obtained in the R-bulk, S-bulk, IR62266 and HCS were 46.08 million, 56.16 million, 58.48 million and 59.87 million, respectively, corresponding to 6.89 Gb, 8.39 Gb, 8.74 Gb and 8.95 Gb (Table 1). The average sequencing depths of R-bulk, S-bulk, HCS and IR62266 were 18.13, 22.10, 23.00 and 23.57, respectively. Based on the read alignments to the Nipponbare reference genome, the proportions of aligned reads in R-bulk, S-bulk, HCS and IR62266 were 97.14%, 97.21%, 97.25% and 97.78%, respectively, covering 91%, 93%, 94% and 92% of the rice genome (Table 1).

### 3.3. QTL-Seq Analysis

**Figure 2:**
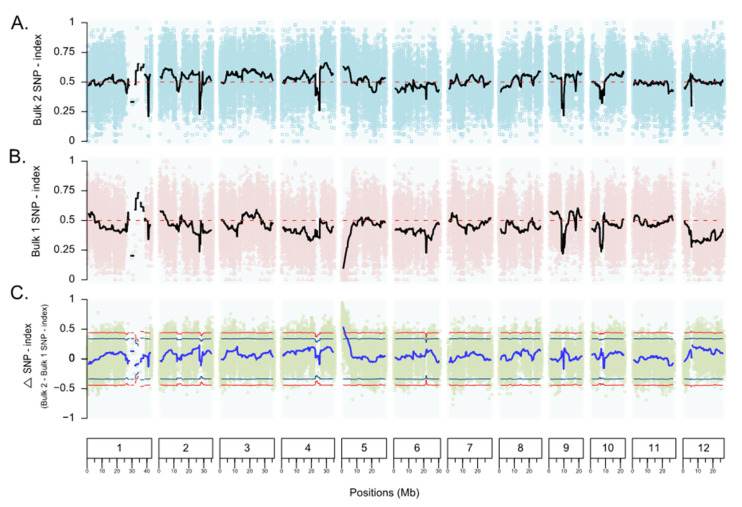
Plots of the SNP index of the R-bulk and S-bulk, and the ∆(SNP index) across 12 rice chromosomes. (**A**) Plots of the SNP index in the S-bulk (blue dots). (**B**) Plots of the SNP index in the R-bulk (pink dots). (**C**) Plots of the ∆(SNP index). Sliding window plots of the average SNP index with a window size of 1 Mb and 10 kb increments are shown as black lines in (**A**,**B**) and as blue lines in (**C**). The green and red line pairs in (**C**) represent the 95% and 99% confidence intervals, respectively.

**Figure 3:**
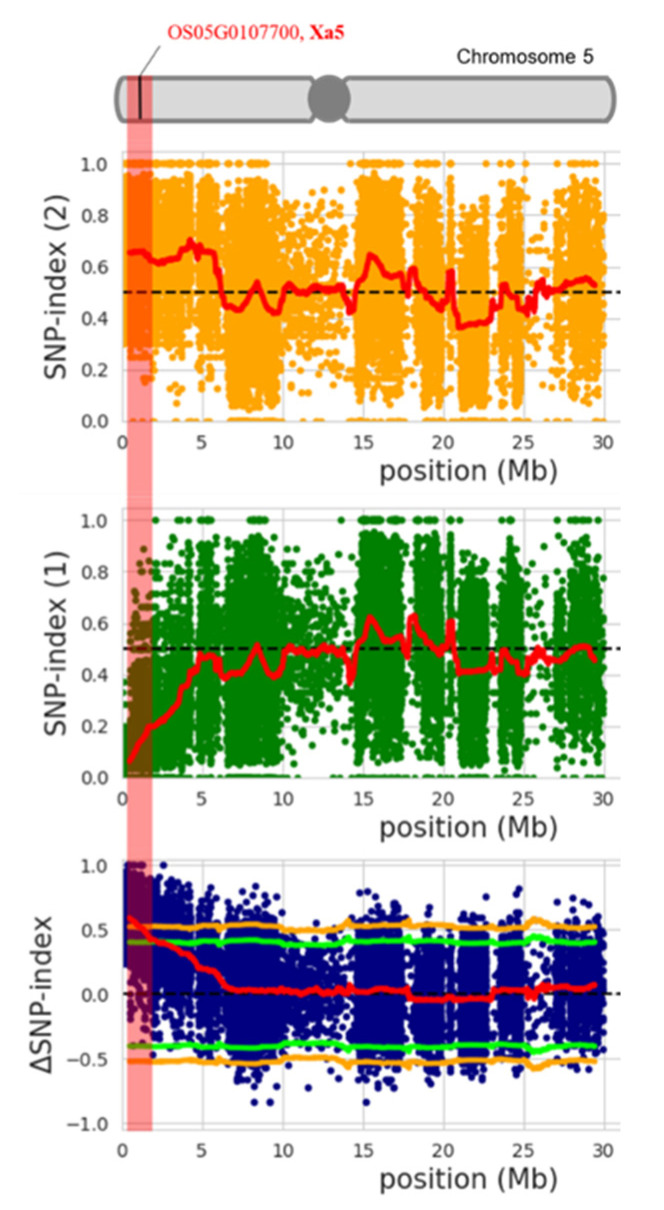
SNP index plots between the S-bulk (top; orange dots) and R-bulk (middle; green dots), and plots of the ∆(SNP index) (blue dots) on chromosome 5 showing the genomic region with different SNP indices in two bulks. The identified region encompassing *xa5* is highlighted.

The SNP variants used in the QTL-seq analysis were the common SNPs identified in both the R-bulk and S-bulk based on read mapping against the parent genome IR62266. Originally, 264,876 SNPs were identified in the two bulks based on a read support criterion of at least five reads (Table 2). However, to obtain a robust result, a read support criterion of 15 reads was applied to filter the initial set of SNPs. As a result, 61,191 SNPs were detected with high confidence from the 12 chromosomes and subjected to the SNP index calculation (Table 2). We also calculated the ∆(SNP index) by subtracting the SNP index values in the R-bulk from those in the S-bulk based on the moving windows of average SNP indices of SNPs located within a 1 Mb region and 10 kb increment. Then, we plotted the ∆(SNP index) across the 12 rice chromosomes to identify the genomic regions of *Xoc* resistance (Figure 2). As a result, a candidate genomic region was significantly identified toward the beginning of chromosome 5, where the average ∆(SNP index) exceeded the 99% confidence interval (Figure 2; Figure 3). SNPs with a ∆(SNP index) of up to 0.90 were identified at a position of 0.44 Mb located within the *xa5* gene (5:437013-443270) (Figure 3; Table 3). 

### 3.4. Development of a Kompetitive Allele-Specific PCR (KASP) Marker for BLS Resistance and Validation

**Figure 4:**
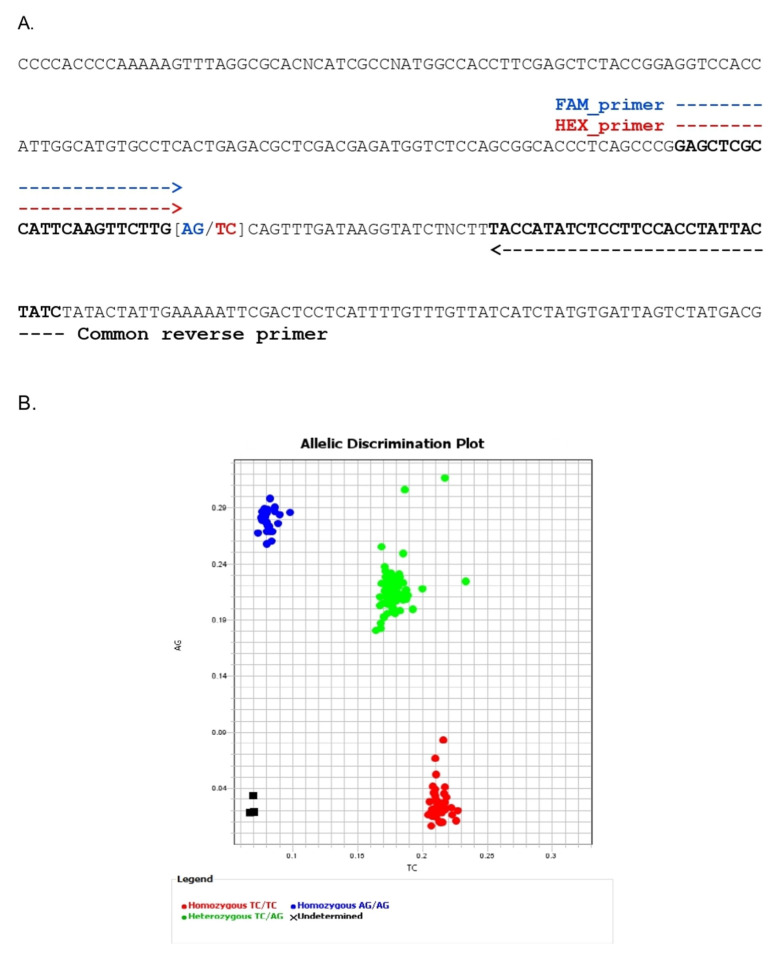
The *xa5*-KASP marker’s development and validation. (**A**) Details on the locations of the selective KASP primers (FAM_primer and HEX_primer) and the common reverse primer. (**B**) Allelic discrimination plots of the PCR results of the *xa5*-KASP markers genotyped in the F_2_ population. Red dots represent the homozygous TC/TC genotype, blue dots represent the homozygous AG/AG genotype and green dots represent the heterozygous genotype.

Based on the results of QTL-seq analysis, *xa5* was identified as a candidate resistance gene for BLS. We then developed a KASP marker (*xa5*-KASP) based on the consecutive SNPs (AG/TC) at positions 437,499–437,500 (Table 4; Figure 4). These two SNPs are located on exon 2, where the substitution of TC by AG causes an amino acid change from Val-39 to Glu-39. IR62266 contains AG, while HCS contains the allele TC at this position. To validate the efficacy of the marker in detecting the resistance of BLS, we developed six F_2_ populations from the crosses of three donors (IR62266, MNTK75 and DV85) and two recipients (RD47 and HCS). All three donors contained the resistance gene *xa5.* Totals of 450, 461, 441, 453, 358 and 441 F_2_ individuals from the crosses (IR62266 × HCS), (MNTK75 × HCS), (DV85 × HCS), (IR62266 × RD47), (MNTK75 × RD47) and (DV85 × RD47), respectively, were used to evaluate resistance to BLS after inoculation by the *Xoc* isolate 2NY2-2, which is another representative of Thai *Xoc*, and which was previously classified in a different group from isolate SP7-5 [30,36]. We then selected 48 plants with disease scores ranging from 1 to 5, and 48 plants with a disease score of 9, in each population for genotyping with the KASP marker. The results show that most of the individuals with disease scores ranging from 1 to 5 contained the homozygous genotype (AG/AG), whereas those with a disease score of 9 contained either the heterozygous genotype (AG/TC) or the homozygous genotype (TC/TC) (Figure S2). We performed a marker–trait analysis between the *xa5*-KASP marker genotypes and the BLS disease scores based on the 96 selected individuals in each of the six F_2_ populations. The results show that the phenotypic variance explained (PVE) values for the KASP marker in the six F_2_ populations ranged from 59.04% to 70.84% (Table 5). The highest PVE value was found in the F_2_ population of MNTK75 × HCS, and the lowest PVE value was found in the F_2_ population of MNTK75 × RD47. It is noteworthy that the PVE values for the *xa5*-KASP marker were relatively lower in the populations in which RD47 was used as the susceptible parent than in those in which HCS was used as the susceptible parent.

## 4. Discussion

Bacterial leaf streak (BLS), caused by the bacterium *Xanthomonas oryzae* pv. *oryzicola* (*Xoc*), is one of the major threats to rice production. In the absence of highly resistant rice varieties, it is difficult to control the disease [3]. Although the disease is known to be one of the greatest devastators of the crop, knowledge of the genes controlling *Xoc* resistance is not very advanced. BLS resistance is considered a quantitative inherited trait because the degree of resistance varies continuously, and ranges from highly resistant to highly susceptible [37,38,39]. The first mapping of the quantitative trait loci (QTL) controlling BLS resistance in rice was reported by Tang et al. [9]. Most of the mapped QTLs showed positive additional effects, indicating that the alleles from the susceptible parent acted to increase BLS susceptibility [9]. To date, more than 20 QTLs have been identified through traditional QTL mapping and genome-wide association studies (GWAS) [9,10,11,30,40]. In addition, a recessive gene *bls1* in wild rice (*Oryza rufipogon*) [41], and the *Xo1* gene, which encodes a putative receptor, have been shown to confer qualitative resistance against *Xoc* from Africa [42].

Most QTLs and genes associated with *Xoc* have been studied in Asian *Xoc* strains, especially from the Philippines and China, and in some African strains [9,10,11,12]. However, QTLs conferring resistance to Thai *Xoc* strains have rarely been reported. The isolates of *Xoc* in Thailand have previously been classified into seven lineages [36]. Five representative *Xoc* isolates from five lineages have been used to identify resistance QTLs using a GWAS method [30]. In this study, we aimed to identify BLS resistance QTLs with a strong effect that can be used in rice breeding programs for BLS resistance to Thai *Xoc* strains. We applied a QTL-seq method to rapidly identify the genomic region associated with resistance to a Thai *Xoc* isolate (SP7-5) based on an F_2_ population. The QTL-seq approach involves bulk-segregant analysis (BSA) and whole-genome resequencing to discover genomic regions associated with the trait by comparing the SNP index in the two bulks with extreme phenotypes. The Δ(SNP index) and a permutation test are used to derive a null model to define significant candidate QTL regions [14]. The power of whole-genome resequencing to discover high numbers of SNPs holds promise for uncovering and improving the detection of target chromosomal regions harboring causal mutations [43]. In general, the QTL-seq approach is faster and more effective than conventional QTL mapping [14]. However, the resolution of QTL-seq depends on the amount of recombination detected in the population and the number of individuals available for the bulks [44]. In recent years, this approach has been used to accelerate the identification of candidate genes for multiple traits in rice [14,15,16,17,18,19,20,21] and other crops [22,23,24,25,27,28,29]. This approach does not require the genotyping of the entire mapping population with genome-wide markers. Only two samples from bulks with extreme phenotypes need to be sequenced. The QTL-seq method is considered an effective method for identifying markers most closely associated with the trait [14]. 

In this study, we used an F_2_ population from a cross between IR62266 and Homcholasit (HCS) to identify a QTL for BLS resistance to a Thai *Xoc* isolate. The resistant parent, IR62266, is highly resistant to both BLS and BLB. This rice cultivar has been used as a source of broad-spectrum resistance to BLB in Thailand [34,35]. The *Xoc* isolate SP7-5 was used as a representative of Thai *Xoc* to inoculate the F_2_ population in the QTL-seq analysis because it clearly exhibits the distinct phenotype of BLS in both parents. In this study, BLS resistance screening was conducted at the seedling stage in the greenhouse (75% relative humidity) using a spray method that has been shown to be effective for the BLS inoculation of early-stage rice plants [30]. The disease was evaluated by observing the severity of the lesion on the leaf, with ratings from 1 to 9 indicating resistance and susceptibility, respectively. The BLS disease scores among the 433 individuals in the F_2_ population were bimodally distributed. The number of plants with a susceptible phenotype was greater than those with a resistant phenotype in this population, suggesting that BLS resistance may be controlled by a few recessive genes. Our QTL-seq results based on this F_2_ population detected only one significant region on chromosome 5, which was strongly associated with BLS resistance. Since the averages of ∆(SNP index) peaked at 0.44 Mb on this chromosome and the SNP position with the highest ∆(SNP index) was found within gene *xa5* (Os05g0107700), we considered *xa5* as the candidate gene for this identified region. The gene was found to have a function in transcriptional activation, and to correspond to the form of the protein present in the rice strain susceptible to *X. oryzae;* it was also found that the substitution of Glu-39 for Val-39 in the rice variety IRBB5 confers resistance to BLB [45]. The same amino acid substitution has also been proposed to increase the resistance of BLS, which has been verified by the RNA interference (RNAi) of *Xa5* [46,47]. In addition to this study, a previous GWAS study reported that the resistance QTL on chromosome 5 encompassing *xa5* confers resistance to four different Thai *Xoc* isolates [30]. Therefore, our study provides additional support, verifying that *xa5* is a potential candidate gene conferring plant resistance to BLS. 

We also developed a KASP marker based on *xa5* and validated the association between the marker and BLS resistance to *Xoc* isolate 2NY2-2, another Thai *Xoc* isolate, in six different F_2_ populations developed based on three donors (IR62266, DV85 and MNTK75) and two recipients (HCS and RD47). With the high phenotypic variance explained (PVE) by these validating populations, the marker could be effectively used in marker-assisted selection (MAS) breeding for BLS resistance. It should be noted, however, that the degree of resistance may vary from population to population. A similar marker specific to SNPs on *xa5* has been developed and used for bacterial leaf blight (BLB) resistance [48]. In this study, we have clearly demonstrated that the marker specific for the same variants can be effectively used for resistance to bacterial leaf streak (BLS) in rice. The *xa5* gene has been used with other resistant (R) genes in rice breeding programs for BLB resistance [49]. According to our results, *xa5* could be suggested as a gene with high potential for BLS resistance. However, qualitative disease resistance (monogenous) usually follows race-specific resistance and quickly degrades due to the rapid evolution of new pathogen races [50]. Alternatively, combining multiple major genes may help to achieve more durable resistance to multiple pathogen races. Several resistance genes have been incorporated through marker-assisted backcrossing (MABC) or conventional backcrossing, and resistance breeding has helped protect rice from pathogen attack [51]. However, molecular breeding via MAS and MABC is inefficient for quantitative traits controlled by many low-effect genes [52]. Alternatively, genomic selection (GS) may be used for the genetic improvement of the complex traits controlled by many genes, each with minor effects [53]. Recently, Merrick et al. showed that they could use GS to predict disease resistance and accumulate beneficial alleles for durable disease resistance [54]. GS models help select cultivars with long-term quantitative resistance by accumulating beneficial alleles, as well as those with disease resistance under conditions that do not favor disease occurrence, which is necessary for phenotypic selection. Genomic selection has paved the way for genomic-assisted breeding (GAB), and could become a promising technique for accelerating the breeding cycle for crop improvement [55,56].

## 5. Conclusions

QTL-seq analysis based on the F_2_ population (IR62266 × HCS) successfully identified a region on chromosome 5 associated with resistance to BLS caused by a Thai *Xoc* isolate. The gene *xa5* was suggested as a strong candidate gene for BLS resistance. The results of this study may be useful for further investigation of the molecular mechanism of the resistance to BLS in rice. The marker, *xa5-KASP*, has been shown to be effective for the selection of BLS resistance, and suitable for use in rice breeding programs.

## Figures and Tables

**Table 1:** Summary of the Illumina sequencing data and mapping statistics of the parental lines and the resistant (R) and susceptible (S) bulks.

Sample	No. of Clean Read (Million)	Clean Base (Gb)	Read Alignment (%)	Genome Coverage (%)	Average Depth
R-bulk	46.08	6.89	97.14	91	18.13
S-bulk	56.16	8.39	97.21	93	22.10
HCS	58.48	8.74	97.25	94	23.00
IR62266	59.87	8.95	97.78	92	23.57

**Table 2:** Chromosome-wise distribution of single-nucleotide polymorphisms (SNPs) between the two bulks.

Chromosome	Length (bp)	Total Number of SNPs (Depth ≥ 5)	Selected SNPs (Depth ≥ 15)
1	43,270,923	26,917	6289
2	35,937,250	28,870	7161
3	36,413,819	26,643	6837
4	35,502,694	23,729	5391
5	29,958,434	26,619	6563
6	31,248,787	20,645	4985
7	29,697,621	24,758	5423
8	28,443,022	20,409	4534
9	23,012,720	12,899	2564
10	23,207,287	14,167	3000
11	29,021,106	21,047	4394
12	27,531,856	18,173	4050
*Total*	373,245,519	264,876	61,191

**Table 3:** Summary of the genomic region associated with resistance to bacterial leaf streak.

Chr.	Position	p99	p95	SNP Index R-Bulk	SNP Index S-Bulk	∆(SNP Index)	Candidate Gene
5	0.44	0.46	0.36	0.00	0.90	0.90	*xa5* (Os05g0107700)

**Table 4:** List of the primers of the *xa5*-KASP marker.

KASP Marker	Primer Name	Sequence (5′-3′)
xa5-KASP	FAM_primer	GAGCTCGCCATTCAAGTTCTTGA
	HEX_primer	GAGCTCGCCATTCAAGTTCTTGT
	Common R-primer	GATAGTAATAGGTGGAAGGAGATATGGTA

**Table 5:** Single-marker analysis of the *xa5*-KASP marker on the 6 populations with *Xoc* isolate 2NY2-2.

Cross	Generation	Total Sample	Tested Sample	PVE (%)	*p*-Value	*Xoc* Isolate
IR62266 × HCS	F_2_	450	96	66.62	<0.001	2NY2-2
MNTK75 × HCS	F_2_	461	96	70.84	<0.001	2NY2-2
DV85 × HCS	F_2_	441	96	69.05	<0.001	2NY2-2
IR62266 × RD47	F_2_	453	96	66.42	<0.001	2NY2-2
MNTK75 × RD47	F_2_	358	96	59.04	<0.001	2NY2-2
DV85 × RD47	F_2_	441	96	65.14	<0.001	2NY2-2

## Data Availability

The data supporting the conclusions of this article are included within the article and its additional files.

## Supplementary Materials

The following are available online at https://www.mdpi.com/article/10.3390/genes12101587/s1, Figure S1: The scale 1–9 of disease scoring on infected leaves, Figure S2: Bar plots of bacterial leaf streak (BLS) scores of F_2_ individuals from six populations classified by genotypes, Table S1: The BLS scores of the parents and 433 F_2_ plants.
