# Supplementary material for: Identification and Validation of a QTL for Bacterial Leaf Streak Resistance in Rice (Oryza sativa L.) against Thai Xoc Strains
Source: Genes (Basel). 2021 Oct 9;12(10):1587. doi: 10.3390/genes12101587 (PMC8535723; doi:10.3390/genes12101587)
Supplement: Supplementary file 1 [file genes-12-01587-s001.zip › Figure S2 - Bar plots of bacterial leaf streak (BLS) scores .pdf]

A. RD47 x MNTK75

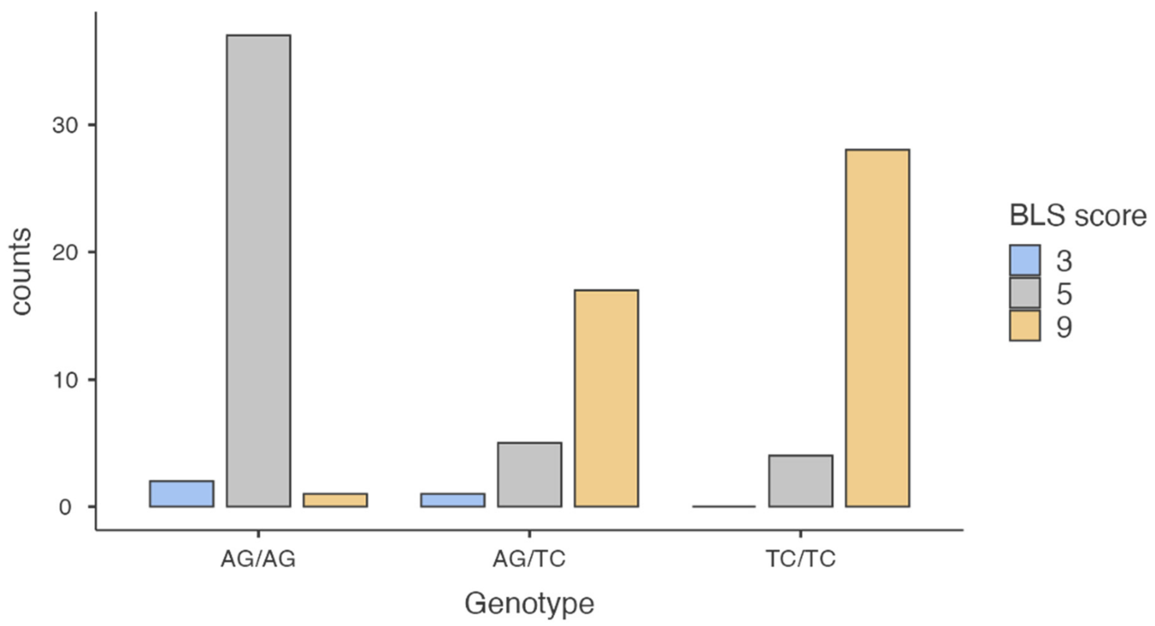

B. RD47 x IR62266

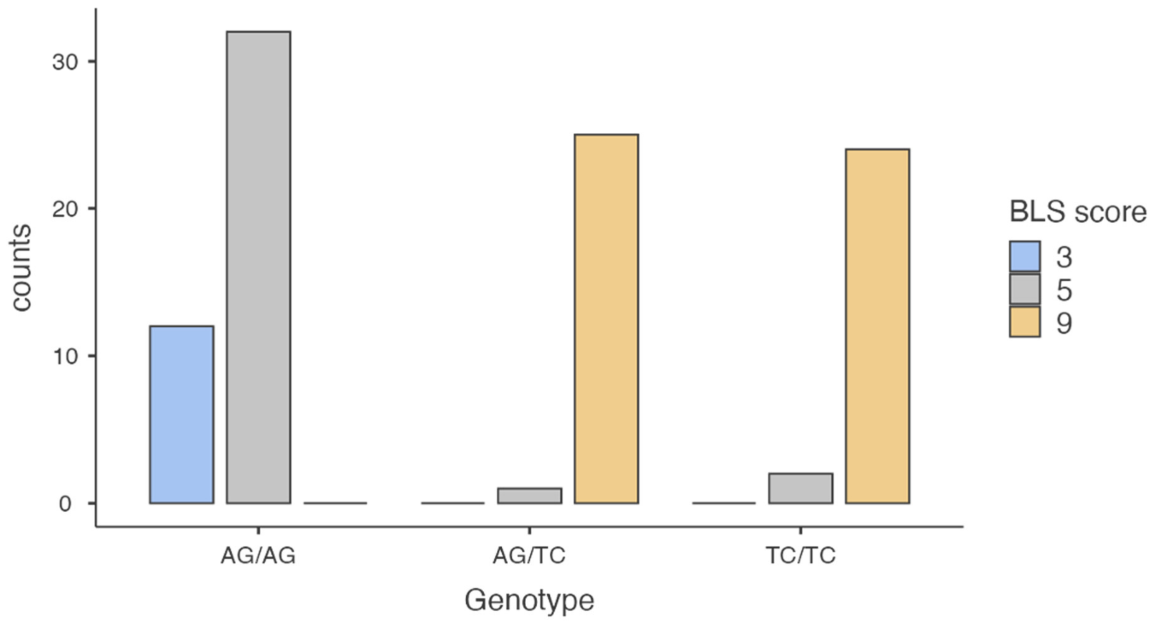

C. RD47 x DV85

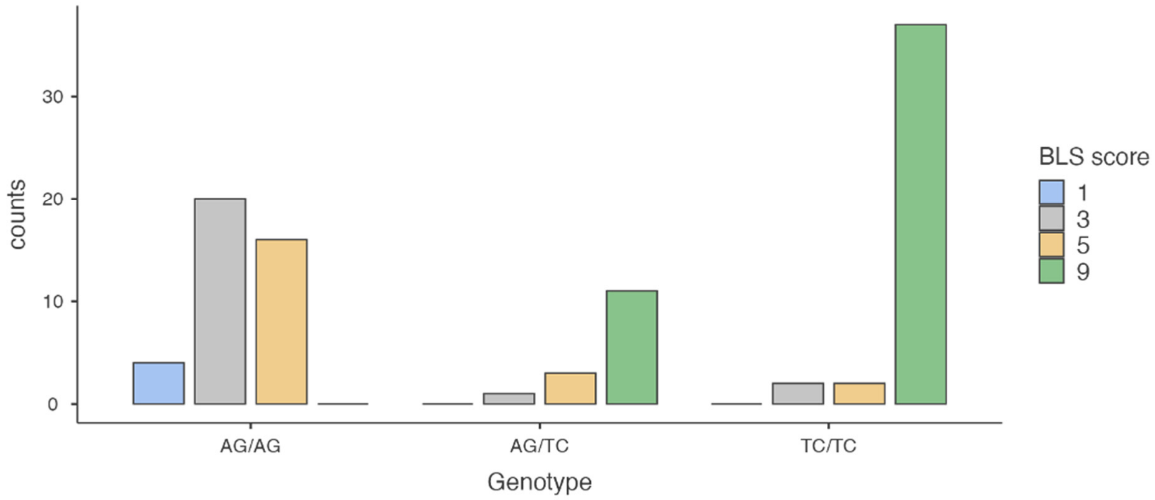

D. HCS x MNTK75

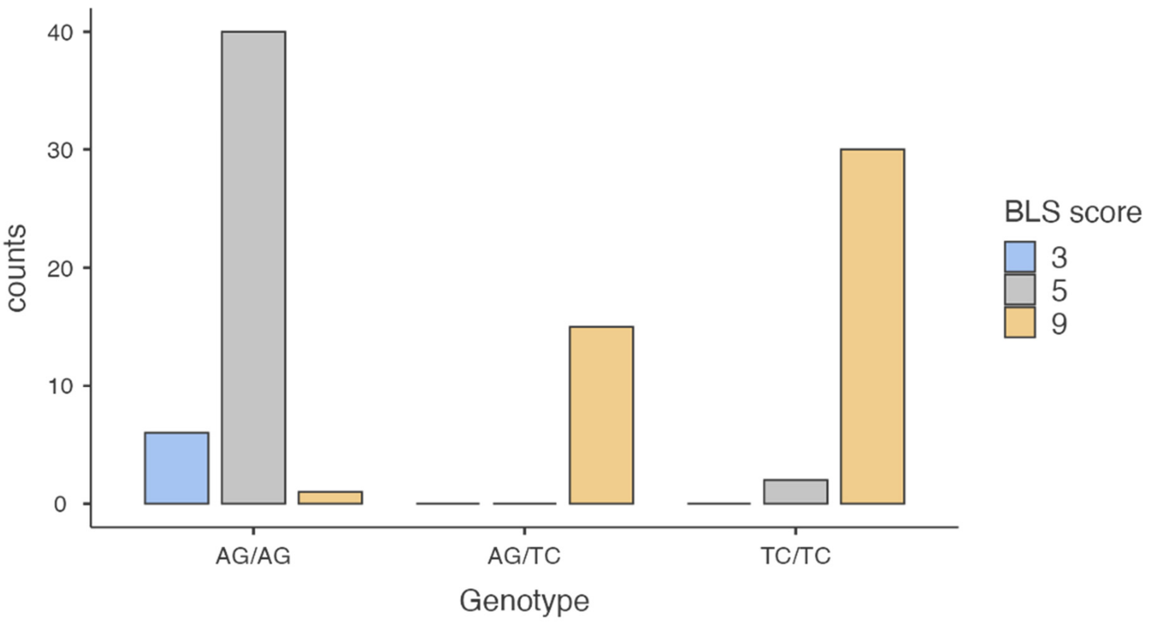

E. HCS x IR62266

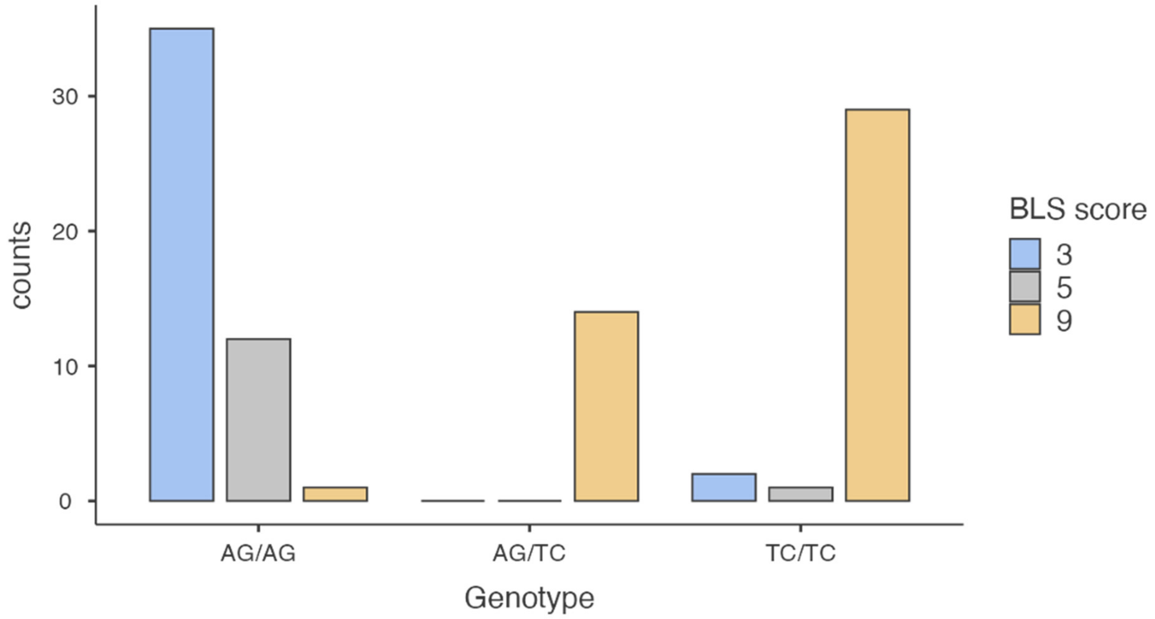

F. HCS x DV85

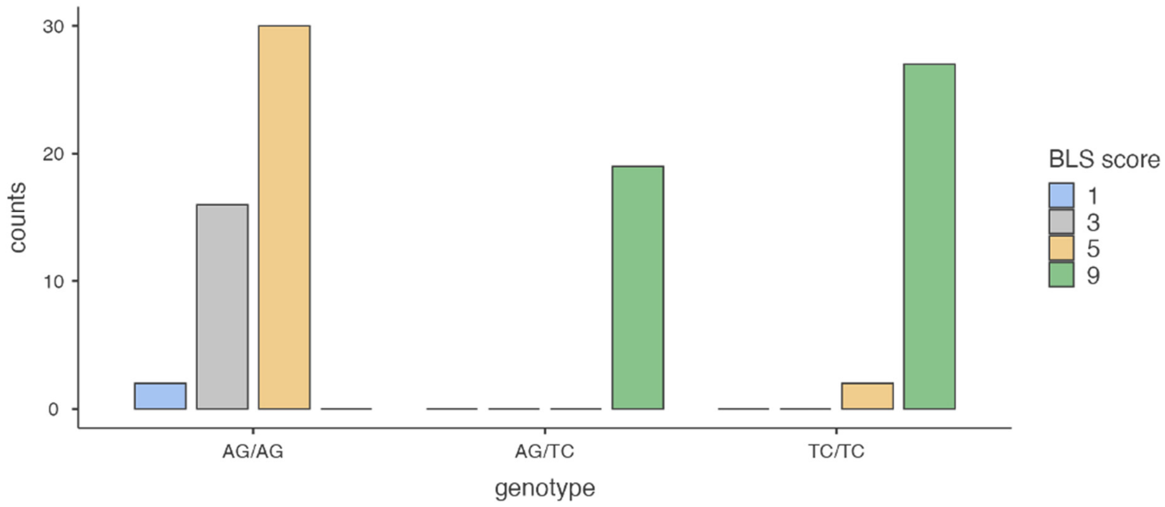

Figure S2. Bar plots of bacterial leaf streak (BLS) scores of F2 individuals from six populations classified by genotypes.
