# Supplementary figures and images for: Identification and Validation of a QTL for Bacterial Leaf Streak Resistance in Rice (Oryza sativa L.) against Thai Xoc Strains
Source: Genes (Basel). 2021 Oct 9;12(10):1587. doi: 10.3390/genes12101587 (PMC8535723; doi:10.3390/genes12101587)

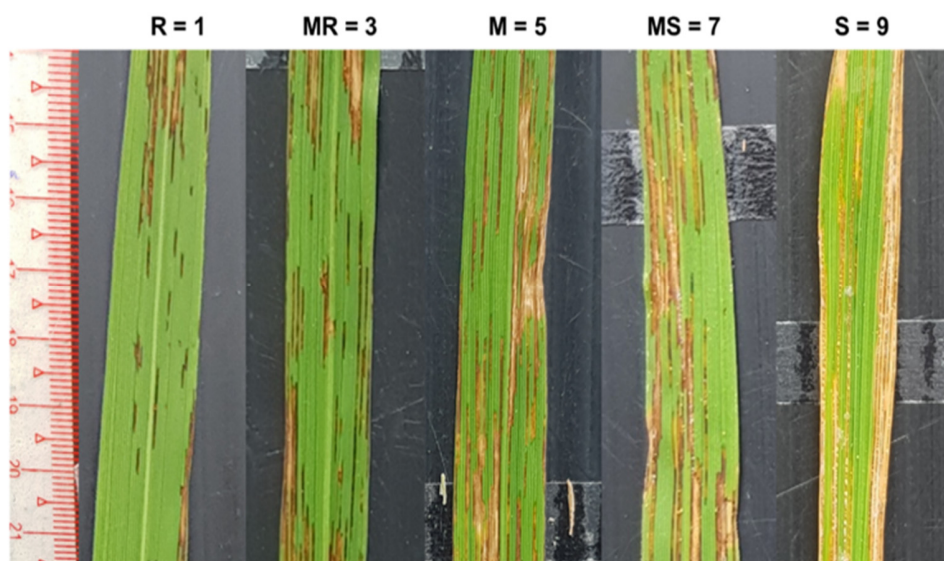

Figure S1. The scale 1–9 of disease scoring on infected leaves, (1 = resistant, 9 = susceptible)

Supplement: Supplementary file 1 [file genes-12-01587-s001.zip › Figure S1 - scoring 1-9.pdf]
